# Acceptability of digital vending machines to improve access to sexual and reproductive health in Brighton, UK: a qualitative analysis

**DOI:** 10.1136/bmjph-2023-000598

**Published:** 2024-05-02

**Authors:** Syra Dhillon, Rhys D Wenlock, Gillian Louise Dean, John Mear, Richard Cooper, Jaime H Vera

**Affiliations:** 1 University of Brighton, Brighton, UK; 2 University Hospitals Sussex NHS Foundation Trust, Worthing, UK; 3 Global Health and Infection, Brighton and Sussex Medical School, Brighton, UK

**Keywords:** HIV, public health, communicable disease control

## Abstract

**Introduction:**

Sexual health remains a public health priority and relies on widely available testing to enable prompt diagnosis and treatment. Technology-based approaches to distribute tests have potential to increase access and enable prompt diagnosis and treatment. We evaluated the acceptability of vending machines (VMs) to distribute HIV self-test (HIVST) and sexually transmitted infection (STI) self-sample kits, from the service user and stakeholder perspective.

**Methods:**

Six VMs were placed across Brighton and Hove (UK) in publicly accessible locations. After use, individuals received a text with an online questionnaire link. Participants completing the questionnaire were invited to a semistructured interview. Stakeholders were staff on sites where the VM was placed. Data analysis took place on NVivo, using a thematic approach.

**Results:**

12 users completed the interview. 42% within the age 18–25 years with equal male and female identifying distributions. 33% were heterosexual, 25% homosexual and 33% bisexual. VM acceptability was high with anonymity and instant access as main benefits. Some participants expressed concern that the public location of the VM may deter others from using it. Participants found the HIVST mouth swabs were acceptable, although there was concern over accuracy. Participants welcomed being able to access comprehensive sexual health screening through a VM. Five stakeholders completed the interview. There was recognition that a publicly visible VM led to positive sexual health conversations between service users and staff. There were initial issues with restocking and machine hardwiring.

**Conclusions:**

VMs to distribute HIV and STI testing kits is acceptable to service users and stakeholders. The main reported benefits are increased confidentiality, privacy and immediate access. Further education referencing the accuracy of the HIVST mouth swab may alleviate concerns. From a stakeholder perspective, the pathway is beneficial and the role of community champions to reduce stigma is favourable.

**WHAT IS ALREADY KNOWN ON THIS TOPIC**

**WHAT THIS STUDY ADDS**

- This study shows that digital vending machines (VMs) are an acceptable method of distributing both HIV self-test and STI self-sample kits to the general population from both the service user and stakeholder perspective. The machines are convenient, easy to use and confidential.

**HOW THIS STUDY MIGHT AFFECT RESEARCH, PRACTICE OR POLICY**

- The VMs are an acceptable adjunct to improve access to testing and current services. Use in clinical practice is beneficial to both service users and healthcare stakeholders.

## Introduction

 Globally, there are approximately 1 million new sexually transmitted infection (STI) diagnoses each day.^1^ Given the asymptomatic nature of most STIs and early HIV infection, there is a significant focus on regular screening to detect subclinical cases. Reducing transmission will lead to a reduction in morbidity and healthcare-related costs.^2^ While testing campaigns have traditionally focused on ‘high-risk’ groups, there is recognition that women, who comprise one-third of those living with HIV, have been overlooked in the HIV and STI narrative.^3^ In 2021 in the UK, for the first time in 10 years, there were more new HIV diagnoses among heterosexuals (49%), compared with men who have sex with men (MSM) (45%).^4^ The HIV and STI demographic has widened, and the screening provisions need to reflect this.

Access to sexual and reproductive health (SRH) is limited globally due to individual factors (risk perception, motivation if asymptomatic), social factors (stigma, cost) and health system factors (testing availability, laboratory capacity). In the UK, most testing takes place in SRH clinics, in general practice (GP) or a kit is ordered online to conduct at home. There are two methods of testing, STI self-sampling (STISS) and self-testing (for HIV only) (HIVST). STISS (for STIs and HIV) enables individuals to collect swabs, urine and a small blood sample and return them via post to the laboratory for analysis. Routine tests diagnose chlamydia, gonorrhoea, syphilis and HIV.^5^ Results are communicated 1 week later via short message service (SMS) or phone call by local SRH services. HIVST allows individuals to obtain either a finger-prick blood sample or mouth swab which gives a result in 20 min. A reactive result must be confirmed with a laboratory test. UNAIDS has advocated for the use of self-testing as part of their FastTrack campaign.^6^

In the UK, the barriers to in-person testing include time and cost spent travelling to the clinic and in the waiting room, risk of seeing someone you know and perceived or actual stigma from healthcare professionals (HCPs).^7 8^ The digitalisation of health services has increased in recent years and has the potential to reduce costs both operationally and by requiring fewer HCPs.^9^ In the UK in 2022, 39% of people used online services to test,^10^ demonstrating an acceptability and demand for home-based testing. However, it requires the user to have a home address, which excludes the homeless or those unwilling to receive a test kit through the post.

To overcome some of these barriers, a vending machine (VM) was installed in 2017 in Brighton and Hove (B&H) (UK) in an MSM sauna. It only distributed HIVST. Implementation analysis demonstrated a significant increase in testing uptake, compared with data from outreach workers testing sauna-goers in-person at the same venue.^11 12^ In this study, 94% of questionnaire respondents stated they would use the VM again. The primary advantage reported by sauna VM users in the interviews was increased confidentiality, convenience and reduced embarrassment. However, the main concern was that the availability of the VM would displace routine SRH screening and reduce testing frequency which would be counterproductive. Following this, a second-generation VM was developed, codelivering HIVST and STISS. The VMs aim to normalise testing by providing convenience, confidentiality and privacy while empowering individuals who may not feel comfortable with traditional testing methods.

Similar studies have looked at VMs in improving access to testing.^11 13 14^ While they found the intervention to be acceptable, they only evaluated HIVST kits and the majority are targeted towards MSM. However, as identified above, there is a gap in the literature and a need to evaluate the acceptability of VMs for both test type kits to the general population. A study in Italy describes how acceptability of VMs to dispense condoms differs between males and females, highlighting the need for further research in this area.^15^ There have been no studies identified that explore the stakeholder views on VMs.

This study aims to investigate the acceptability of obtaining HIVST and STISS from VMs located in community settings from the service user and stakeholder perspective.

## Methods

### Study design

This paper is the partial publication of a mixed methods study that we have split into two papers due to the high volume of valuable data yielded. The quantitative aspect has been published.^16^

We conducted an exploratory qualitative study with VM users and stakeholders between May and September 2022. VMs, manufactured by Aeguana,^17^ are in six locations in B&H: a community centre reception area, library foyer, GP practice waiting room, pharmacy shop floor, MSM sauna entrance and on campus of the University of Sussex student union. There is a seventh machine in an adult entertainment shop which only dispenses HIVST.

Users are presented with a digital interface and prompted to answer demographic questions (age, gender, sexuality, place of residence and time since last HIV/STI test). There is a minimum age of 18 for use. Following this, users select their test type. All the STISS kits contain a self-taken blood sample bottle and swabs/pots depending on gender and sexuality.^18^ The test kit includes a QR code which links back to the local SRH service.^19^

VM users are required to input a mobile phone number to obtain a four-digit access code by SMS. When the code is entered, the kit of choice is dispensed. The purpose of the code is to prevent the same user from obtaining several tests and potentially selling them for secondary profit. The encrypted mobile phone number can only be used once over a 14-day period and is stored on a secure server.

### Participant recruitment

In the same SMS as the four-digit access code, is a message and link to an online questionnaire that invites VM users to complete a survey on their views in exchange for a high street voucher (online supplemental material 1). At the end of the survey, users could tick a box to indicate interest in subsequent interview. These participants were asked to provide an email address, so the investigators could provide the PIS/CF (online supplemental materials 2 and 3) and organise the interview.

Stakeholders included volunteers working independently from the VM team at each location responsible for the machine and answering any questions that users might have. Stakeholder participants who consented to interview were offered a high street voucher.

This study aimed to recruit 15 participants (10 users, 5 stakeholders), consistent with the previous study that took place in Brighton to assess the HIVST machines in 2017^11^

### Data collection

All participants gave informed consent. The online questionnaire collected demographic data. Interview guides (online supplemental materials 4 and 5) developed by the study team were used to structure the interview with open-ended and probing questions. Key themes included motivation to test, testing history, acceptability of testing methods and venues and acceptability of the VM testing pathway. In-depth semistructured 30-minute interviews were conducted either online via Microsoft Teams (n=15) or face-to-face (n=2, took place in private rooms at VM sites) depending on participant preference.

All interviews were conducted by SD who is a female junior doctor with a particular interest in SRH and with MSc level training in the theory of qualitative data collection and analysis. SD had only minimal experience in practically conducting qualitative work at this stage. SD’s only prior relationship with the participants was arranging the interview schedule via email. It was explained to participants at the start of the interview that SD was part of the research team evaluating the VMs.

Interviews were audio and visual recorded, transcribed verbatim and checked for accuracy. Field notes were taken during the interview and destroyed confidentially. No repeat interviews took place. No one else was present except the participant and researcher.

### Data analysis

Demographic characteristics of participants were analysed using descriptive statistics using SPSS V.28. Data analysis of interview transcripts annotated with field notes used NVivo software V.1.6.2 and was analysed using thematic analysis, informed by Braun and Clarke^20^ to identify themes related to the acceptability of VMs.

SD, RDW and JHV familiarised themselves with the dataset. SD and RDW completed the first stage of coding independently. This involved the systematic labelling into broad themes to capture the important features of the dataset. This was amalgamated and discrepancies (31 inconsistencies across 394 references) were discussed in a meeting between SD and RDW to discuss areas of similarity and conflict until a consensus was reached, as well as defining scope and focus of each theme identified. SD and RDW then continued to refine initial themes and finalise assigning themes in ongoing discussions with additional input from JHV until all three authors had signed off on the final codebook (online supplemental material 6). SD wrote up the analysis into a narrative with illustrative quotes, which was reviewed by RDW and JHV.

Missing transcript data occurred once when SD’s laptop did not record a face-to-face interview appropriately. This was mitigated by immediate recall and record of the interview content from memory and field notes and using respondent validation to send the summary to that participant for urgent review.^21^ The participant suggested two additions to the document which SD agreed with and added. There were no disagreements between SD and this participant with regards to contents of reconstructed interview. No other participants were able to review or edit their transcripts for accuracy due to time constraints.

Data were reported using the COREQ checklist (online supplemental material 7).

###  Ethical approval

This study received ethical approval from NHS Health Research Authority (IRAS ID 306738). The study protocol was published online at Sussex Research Online.^22^

## Results

257 people used the VM in the 5-month time period, 34 users completed the online questionnaire and 12 completed the interview (16 consented, 4 did not engage for unknown reasons). 42% were in the 18–25 year age range. Participants were mainly white (83%), with equal male and female identifying distributions. 33% of participants identified as heterosexual, 25% homosexual, 33% bisexual and 8% preferred not to disclose. See table 1 for full participant characteristics. Interview participants were generally matched to the total VM users except fewer heterosexuals and never testers as well as across a limited range of VM venues.

**Table 1:** Demographic data of service users in comparison to total VM users where available

	Interview participants		Total VM users	
	N=12	%	N=257	%
Age (years)				
18–25	5	42	129	50
26–35	3	25	65	25
36–45	3	25	32	12
46–55	0	0	20	8
56+	1	8	11	4
Gender				
Male (including trans-male)	6	50	141	55
Female (including trans-female)	6	50	102	40
Non-binary	0	0	11	4
Not specified	0	0	3	1
Sexuality				
Heterosexual	4	33	122	47
Homosexual	3	25	63	25
Bisexual	4	33	63	25
Not specified	1	8	9	4
Ethnicity				
White	10	83	Not available	
Mixed/multiple	1	8		
Asian	1	8		
Last HIV test				
<3 months	2	17	45	18
3–12 months	5	42	64	25
More than 1 year	2	17	46	18
Never	3	25	102	40
Last STI test				
<3 months	4	33	56	22
3–12 months	5	42	61	24
More than 1 year	1	8	67	26
Never	2	17	73	28
Pre-existing medical condition				
Mental health condition	2	17	Not available	
Learning disability	2	17		
Problematic drug/alcohol use	2	17		
VM location accessed				
Library	8	67	123	48
Community centre	2	17	16	6
MSM sauna	0	0	24	9
Pharmacy	1	8	34	13
University campus	1	8	44	17
GP practice	0	0	16	6

Participants were asked to state their age range from 18 to 25, 26 to 35, 36 to 45, 46 to 55 and 56+, rather than specific age. Percentages are calculated to the nearest round number.

GPgeneral practiceMSMmen who have sex with menSTIsexually transmitted infectionVMvending machine

Five stakeholders were interviewed, four employees from venues (library, GP practice, MSM sauna and adult entertainment shop) and one from the local SRH service. 80% of stakeholders were male.

Three principal themes were identified and are summarised with illustrative quotes in table 2.

**Table 2:** Key themes with illustrative quotes (participant identifier)

Theme	Illustrative quote
1. Perceptions of testing	I would definitely go to the walk in if I was symptomatic. I’d want to be able to talk to someone. It would probably lead to me being able to get treatment for it faster as well. (P17: service user) If it was just a HIV test then it would almost be a little bit like a COVID test where you just do a quick look and see, but you’d still do a full screen. I would still need to go and arrange my full screening so I would end up doubling up my testing. So for me personally, it’s gotta be a full test kit cos then it basically replaces the inconvenient thing of going in [to the sexual health clinic]. (P2: service user) I probably would have said the mouth swab three years ago, but where [COVID-19] lateral flows are so insensitive, I kind of associate swab test being a bit rubbish and so I think the finger prick [HIVST] in my mind was better. So, I’d go for that. (P13: service user)
2. Advantages of HIV and STI VMs	I see the benefit of it. It’s quick and easy and I've got kids, so going into a library and them running off to get books and me typing my number into a vending machine and getting a pack out and then doing it on my time is super simple (P9: service user) I think it’s been viewed as quite positive because it has helped ease some of the pressure on us as well. Obviously we’ve all been very busy. All services are stretched at the moment. So if this means that a patient can have access to the care that they need sooner than that’s great. (S3: stakeholder) You want it to be a place that’s easy to access. The emergency department at the [local hospital] has a lot of people coming through who are misunderstanding the service and turning up when actually what they actually need is sexual health testing. And so rather than people being told to go and book a sexual health appointment, if there was a machine on site that they could use without having to involve hospital staff. (P13: service user)
3. Limitations of HIV and STI VMs	I suppose there’s like there’s a slightly nerve wracking positioning of it being like right on an entrance. So like people are gonna be walking by while you’re kind of there doing it and it’s like less discrete than somewhere else. But equally because it is there, you notice it and you see it so you use it. (P1: service user) With regards to timings, you know because it’s the one in the library for example, you know it’s only available when the libraries open and I suppose that’s a good thing because I'm sure if it was in the street outside it would be vandalised or whatever. (P6: service user) The team that’s putting them through has a lot of other pressures from the other postal testing. And it’s kind of been rolled into another team that was already doing something similar… if we want to be doing more through vending machines, you are gonna need a team. Obviously, it’s quite small [currently]. But if you wanted to push it more, that’s probably what you’d need. (S4: stakeholder)

Participant numbers refer to whole mixed method study numbers which is why N>12. P refers to service user and S refers to stakeholder.

HIVSTHIV self-testSTIsexually transmitted infectionVMvending machine

### Theme 1: perceptions of testing

Principal incentives to test were a desire for safe sexual practice, frequency of sexual activity and perceived risk of sexual encounters. If participants were symptomatic, they would prefer to attend the clinic, so they could be examined and gain information from an HCP (quote 1.1). Two participants (17%) would not have tested if not for the VM.

Opinions of HIVST were positive, with speed, privacy and discretion in testing location quoted by most participants. However, there were concerns about reliability, with greater trust afforded to laboratory testing. Participants were in favour of getting a comprehensive SRH screening from the VM, rather than engaging with traditional services for bacterial STI tests (quote 1.2). It was agreed that including all infections led to the normalisation of testing, particularly among those who had never had an HIV test before. The response to STISS was positive and most participants were familiar with the process.

There was agreement that COVID-19 lateral flow tests (LFTs) have resulted in a greater acceptability of home testing and have improved confidence in conducting HIVSTs independently. Most participants preferred the less intrusive and pain-free feature of the mouth-swab HIVST. However, two participants had concerns over accuracy and drew parallels with the COVID-19 LFTs (quote 1.3).

Stakeholders from venues with a visible VM (ie, in the foyer) agreed that the machine generated positive conversation among customers and staff members, especially about HIV and the importance of regular testing.

### Theme 2: advantages of HIV and STI VMs

Most participants found the VM easy to use and all participants would recommend the intervention. The main positives were that it was free to use, had instant access, and was placed in venues which were frequently attended, providing an opportunistic testing route (quote 2.1). Most participants anticipated that knowing the VM sites would increase testing frequency in future. Privacy was a common motivation for VM use, with anonymity and discretion in the vend as perceived benefits. There was appreciation of how the kit included a QR code, which included communication on broader SRH services, including contraception and in-person clinics. The machine in the library was particularly popular. Staff reported being confident in answering customer questions, signposting to appropriate resources and escalating concerns.

All stakeholders had seen customers interact positively with VMs and found it easy to use without assistance. One participant from a GP clinic in a low socioeconomic area described how their patients can be distrusting of HCPs, so the VM provides an opportunity to access healthcare without requiring face-to-face interaction. There was recognition that the VM eased pressure on their stretched services, providing quicker access to testing for their population (quote 2.2).

All participants suggested locations within B&H for future VMs, including student and social venues, other GP centres/pharmacies, community centres and shopping centres. It was suggested that a VM could be placed at the main hospital emergency department entrance, as a method of diverting away from secondary care services (quote 2.3). In terms of what else the machine could offer, condoms were the most frequently suggested item, followed by period products. Two participants taking regular HIV pre-exposure prophylaxis enquired whether the VM could act as a substitute for attending the clinic for their bi-annual screen, by integrating systems further.

### Theme 3: limitations of HIV and STI VMs

Participants understood the public location of VMs was vital for visibility, footfall and normalisation of testing. However, for the library VM, the location received mixed opinions; there were concerns that the orientation of the machine meant the screen was facing the walkway, where people walking past could see the sensitive information being input (quote 3.1).

The mechanics of the machine occasionally posed an issue, for example, the box could drop down and block the opening. On these occasions’ participants sought assistance from staff on site, who were able to obtain a kit by opening the machine.

Three participants disclosed previous attendance at a VM that had been without stock and that this decreased confidence in the service. Approximately half of the participants thought this would deter them from using the service again in future.

The timings that the VMs are available are dictated by the opening times of the venue. For the sauna, this is a 24/7 provision but for other venues this can be 18:00 at the latest, meaning access may be difficult for those who work full-time or outside the city (quote 3.2). Participants advocated for further venues with longer opening times or suitable signposting to other services that could be reached out of hours. Discussion around how accessible the machine is to disabled people. All participants were able-bodied but three commented on the screen height and whether someone in a wheelchair would be able to use it.

Stakeholders found that on occasions VMs could turn off without obvious reasons and when stakeholders attempted to contact the manufacturer, they were not always responsive. While these issues were referred to as ‘teething problems’, stakeholders were concerned about how machine downtime could negatively affect service users’ perception of the service and reduce future use if they persisted.

Venues felt that it was sometimes difficult to get in contact with an appropriate team member to answer queries. This could mean that the VMs were out of stock for some time. From the SRH clinic’s perspective, the VM service has been integrated into the roles of the chlamydia screening programme, a team with an already high workload, and a staff member warns that prior to expansion, more staff and an extra team would be required to ensure the service runs capably (quote 3.3). This includes packing the kit boxes, restocking the VMs every 2 weeks, sorting and registering the kits on return to the service and acting as a liaison point for venues for queries.

## Discussion

We found that HIVST and STISS kits distributed by VMs was an acceptable method to improve access to SRH services in B&H, and this is the first qualitative study to evaluate the codistribution of both test kit types. The machines are accessible and convenient, although further work on exact positioning, mechanics and venue opening times is important to improve the pathway.

HIVST acceptability in this study was high with confidentiality and speed as the main benefits, consistent with published literature both within the UK and globally.^23 24^ The use of the mouth swab, compared with the finger-prick, was positive, similar to published studies.^25 26^

There were some user concerns about the accuracy of mouth swabs, which was also described by Nangendo *et al* suggesting that some people will still subsequently attend the clinic^27^ to have a formal confirmatory blood test. Our participants discussed this following their experiences with COVID-19 LFTs.

Regarding the codistribution of HIVST and STISS packs, these results were encouraging. Given the same mode of transmission for these infections, it is encouraging that VMs can act as an intervention to tackle common STIs.

The VM was regarded as an acceptable method of kit delivery, with all users indicating they would recommend the service, consistent with previous studies both within B&H and globally, including USA, Japan, China and Australia.^11^,^1428^

Disability has been identified as an independent risk factor for STIs due to health inequities and poor quality sexual education.^31^ The VMs are standing height which may mean some people cannot reach to the full screen height. However, at the time of writing, the VMs can now project a mirror interface onto the user’s phone, which allows access for all users; this would also provide increased privacy when answering the demographic questions, which was highlighted as a concern.

Clinic and postal services are popular in the UK, carrying out 92% of tests^32^ and providing counselling, partner notification and free condoms. Clinics also offer additional specialist services, for example, hepatitis screening and harm reduction. These services are not provided by the VM itself but by a link to online services and the clinic is provided through a QR code. A literature review investigated how decentralised services pose a safeguarding risk as social factors (such as domestic violence) cannot be identified, unlike in clinic.^33^ This emphasises the role of VMs as an adjunct to testing, rather than a mechanism to replace the entire pathway.

This is the first study to discuss feasibility from a community stakeholder perspective and the feedback is encouraging, with room for improvement regarding the technical aspects. All stakeholders felt comfortable engaging with customers to discuss the VM and the aim to improve testing access. It was acknowledged that the VM presence encouraged positive conversation, which is useful in reducing stigma. Therefore, the stakeholders act as community champions to empower individuals, a role that is recognised globally as a successful mechanism in bridging a gap to connect community members, particularly from disadvantaged backgrounds.^34^

It was encouraging to see how the HCP stakeholder recognised that the VM was able to ease resources at the GP surgery, giving patients earlier access to testing. Despite this, there were concerns about sustainability of scale-up from an SRH staff perspective (packing kits, VM restocking) which would need to be explored prior to substantial expansion, including to other healthcare facilities, such as the emergency department.

There are further limitations of this study. First, those who did not use the VM (eg, intervention is not acceptable to them or they did not have sufficient digital literacy) were not incorporated into this study. It would be essential to get the views of this population prior to expanding this service to optimise improving access. Additionally, the VM did not capture what proportion of participants are transgender and also was not accessible to those under 18, both of which are priority groups and could represent an important focus for future research. Finally, the interview uptake overall was low and included fewer heterosexual and never testers than the overall VM users so therefore not necessarily representative and generalisability may be limited.

## Conclusion

This study aimed to explore acceptability of VMs to distribute HIVST and STISS kits from the service user and stakeholder perspective. There are gaps in testing provision which prevent timely diagnosis and treatment. These gaps have historically affected ‘high-risk’ groups but now the shifting demographic means testing strategies to improve access are imperative.

The role of a VM to codistribute HIVST and STISS kits is encouraging with confidentiality and immediate access as the main benefits. More education concerning the HIVST mouth swab is required to counter concerns. From a stakeholder perspective, the pathway is beneficial and the role of community champions to reduce stigma is encouraging.

Further work on operational sustainability with scale-up and targeted focus groups for those who approach the machine and do not use it, as well as heterosexuals, never testers and disabled people to discuss accessibility, are recommended.

## supplementary material

10.1136/bmjph-2023-000598 online supplemental file 1

## Data Availability

All data relevant to the study are included in the article or uploaded as supplementary information.
