# Supplementary material for: Acceptability of digital vending machines to improve access to sexual and reproductive health in Brighton, UK: a qualitative analysis
Source: BMJ Public Health. 2024 May 2;2(1):e000598. doi: 10.1136/bmjph-2023-000598 (PMC11812820; doi:10.1136/bmjph-2023-000598)
Supplement: online supplemental file 1 [file bmjph-2-1-s001.pdf]

1. Online questionnaire

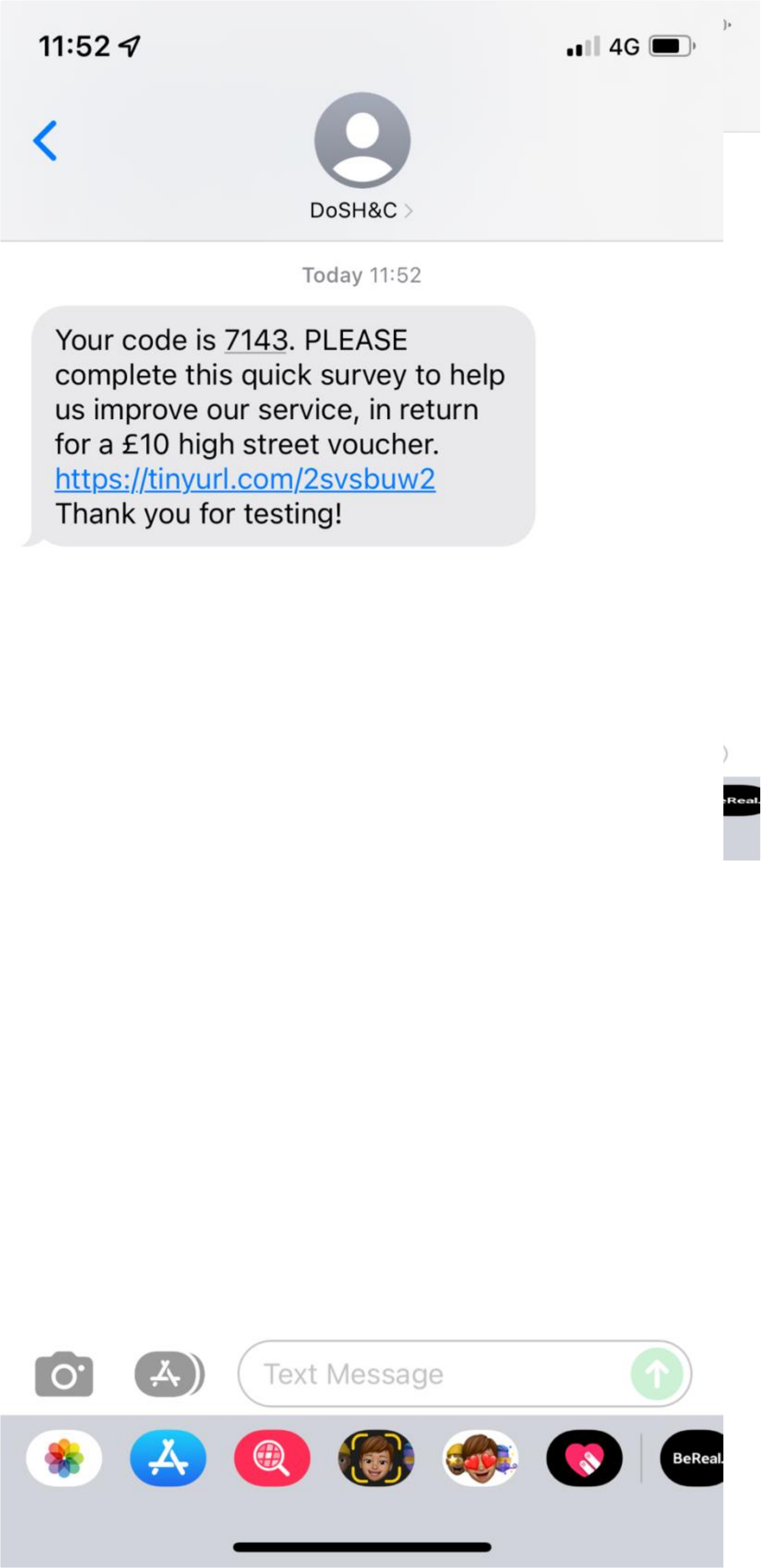

Vending Machine 2022

0% complete

Page 1: Page 1

Thank you for using our NEW vending machine!

Your feedback is very important and will help us understand whether vending machines are a useful way to offer HIV and STI tests. Your answers will help improve services.

We would be grateful if you would fill in this 5 minute ANONYMOUS survey to help improve our services in exchange for a £10 high street voucher. We are no longer running the interview component of this evaluation.

Before you decide whether to answer, it is important that you understand why this project is being done and what it will involve. Please access more information by clicking on the Patient Information Sheet.

1. I have read the patient information sheet above and consent to my data being used in this study. ✱ *Required*

   - ○ Yes

2. What is your age? ✱ *Required*

   - ○ 18-25
   - ○ 26-35
   - ○ 36-45
   - ○ 46-55
   - ○ 56+

3. What is your gender? ✱ *Required*

   - ○ Male (including trans-male)
   - ○ Female (including trans-female)
   - ○ Non-binary
   - ○ Prefer not to say
   - ○ Other

4. What is your ethnicity? ✱ *Required*

- ○ White (includes: English, Welsh, Scottish, Northern Irish, Gypsy, Any other White background)
- ○ Mixed or multiple ethnic groups (includes: White and Black Caribbean, White and Black African, White and Asian, Any other Mixed or Multiple ethnic background)
- ○ Asian or Asian British (includes: Indian, Pakistani, Bangladeshi, Chinese, Any other Asian background)
- ○ Black, African, Caribbean or Black British (includes: African, Caribbean, Any other Black, African or Caribbean background)
- ○ Other ethnic group (includes: Arab, Any other ethnic group)

5. Is English your first language? If no, please state your first language. ✱ *Required*

- ○ Yes
- ○ Other

6. I have sex with... ✱ *Required*

- ○ Men (including trans-men)
- ○ Women (including trans-women)
- ○ Men and women
- ○ Prefer not to say

7. Do any of the following apply to you? ✱ *Required*

- ☐ Visual impairment
- ☐ Hearing impairment
- ☐ Long standing physical health condition
- ☐ Long standing mental health condition
- ☐ Learning disability
- ☐ Problematic drug/alcohol use
- ☐ None of the above

8. Please describe your housing situation. ✱ *Required*

- ○ Securely housed
- ○ Insecurely housed
- ○ Street homeless
- ○ Prefer not to say

9. When was your last HIV test? * *Required*

- Less than 3 months ago
- 3-6 months ago
- 6-12 months ago
- 1-3 years ago
- More than 3 years ago
- Never

10. When was your last STI test? * *Required*

- Less than 3 months ago
- 3-6 months ago
- 6-12 months ago
- 1-3 years ago
- More than 3 years ago
- Never

11. Where did you hear about the vending machine? * *Required*

- Word of mouth
- Advice from GP/healthcare professional
- Saw it in person
- Social media
- Other

12. What services have you used to check your sexual health before? * *Required*

- Ordered online
- Sexual health clinic
- GP
- Pharmacy
- School/college/university
- Vending machine
- I've never tested before
- Other

13. Why did you choose to get a kit from the vending machine? ✱ *Required*

- It feels more confidential / private
- I did not want a test kit coming through the post to my home
- I did not want to visit a sexual health clinic
- I don't have internet access
- I wanted instant access
- It was more convenient
- Other

14. If the machine wasn't here, how would you test for HIV/STIs? ✱ *Required*

- Sexual health clinic
- Postal service
- GP
- Pharmacy
- I wouldn't have tested
- Other

15. How would you rate the user-friendliness of the machine? ✱ *Required*

- Very good
- Good
- Average
- Poor
- Very poor

16. Which location did you use the vending machine? ✱ *Required*

- Brighton sauna
- Jubilee library
- Portland road
- Wellsbourne Centre
- BMECP centre
- Brighton campus
- Sussex campus
- AMEX stadium

17. What do you think of the location of the vending machine? ✱ *Required*

- ☐ It was in a convenient location
- ☐ It was easy to locate
- ☐ I could access the machine without any assistance
- ☐ I had no concerns about my safety when using the machine
- ☐ I had no concerns about my privacy when using the machine
- ☐ Other

18. Would you recommend this service to your friends? ✱ *Required*

- ○ Yes
- ○ No

19. Any further comments?

20. Please enter your email address to receive your £10 high street voucher. ✱ *Required*

21. I am interested in finding out about the results of this study and consent to being contacted with a copy of the results. ✱ *Required*

- ○ Yes
- ○ No

22. I am interested in taking part in a 30 minute interview to discuss my experience, for a further £20 high street voucher. *Optional*

- ○ Yes
- ○ No

2. Interview PIS

Patient Information Sheet – Interview – B&H

Acceptability and feasibility of electronic vending machines for delivering HIV self-testing and STI self-sampling kits: a mixed methods study

Chief Investigator: Dr Jaime Vera
Version 5 16/05/22

You have used a vending machine and we would like to find out more about your experiences of using one.

In this study we will use information from you. We will only use information we need for the research study. We will let very few people know your details, and only if they really need it for the study. Everyone involved in this study will keep your data safe and secure. We will also follow all privacy rules.

At the end of the study we will save some of the data in case we need to check it and for future research. We will make sure no-one can work out who you are from the reports we write. This information pack tells you more about this.

Why is this study being done?
You are invited to take part in an interview for a research study about the use of a vending machine to deliver HIV and STI tests in the community. Before you decide whether to take part, it is important for you to understand why the project is being done and what it will involve. Please take the time to read the following information carefully and feel free to discuss your participation with your family and friends if you wish. Please ask a member of the research team if anything is unclear or you would like more information. This study will make up part of a dissertation for a student researcher in their Global Health MSc.

What is the study aim?
Effective treatment for HIV and STIs are available but relies on initial diagnosis and prevalent testing. We are using vending machines to deliver HIV and STI test kits in five locations in Brighton and Hove and four locations in Bristol, North Somerset and South Gloucestershire to determine whether they provide a suitable avenue to increase access to tests. The study aims to explore the number of HIV and STI tests that are distributed, the demographics of the population using the vending machines and the acceptability and practicality of using vending machines to dispense HIV self-testing kits in the community. This is a novel strategy for kit distribution that was previously implemented via vending machines.

Who can participate?
Adults (18 years and older) who have used the vending machine are invited to interview. It is up to you to decide whether or not to take part.

What is involved?
This will involve a 30-minute interview regarding your experience of the machine and test kits. There are no right or wrong answers - it's your experiences and views we are interested in. We will use this information to do the research. Answers will be anonymous and securely stored. We will not link your name or any identifiable information to your responses and keep all information safe and secure. The interview will be recorded so we can accurately recall what you have said. We will store the tape in a secure and anonymous

way. Answers will be transcribed either internally or by an external service but all information is anonymous and we will not link your name or any identifiable information to your responses.

Do I have to take part?
No. It's up to you. Whether you decide to take part or not will not affect your care through the NHS. Completing the interview is voluntary and you can stop at any time. If the questions are upsetting or if you have any concerns, please ask us. If these concerns persist, please contact your healthcare provider/GP.

How will we use information about you?
We will need to use information from you for this research project. This information will include your:

- Age
- Gender
- Ethnicity
- Sexual orientation
- Contact details
- Additionally it will involve discussions about previous experiences with HIV and STI testing, the role of HIV self-tests, the use of vending machines and experience using the machine.

Recordings and transcripts will be anonymised by allocating a number to each participant in order to track their responses. Data will be exported to SPSS and NVivo for analysis in a password protected file and laptop that only the research team will have access to. People will use this information to do the research or to check your records to make sure that the research is being done properly. People who do not need to know who you are will not be able to see your contact details. We will keep all information about you safe and secure. Once we have finished the study, we will keep some of the data so we can check the results. Personal data will be held for a maximum of six months and research data will be kept for ten years. We will write our reports in a way that no-one can work out that you took part in the study. Data will be shared with sponsor representatives who may see it in identifiable forms for data monitoring purposes. Data will be anonymously deposited in the UK Data Archive for reuse in future research and analysis.

Will my taking part in this study be kept confidential?
Yes. The information you give us will be kept confidential. When processing and storing information, we will comply with the Data Protection Act 2018 to protect your confidentiality. We will only use your phone number and no other personal identifying information to conduct the study. We will follow all privacy rules. At the end of the study we will save some of the data in case we need to check it. By agreeing to take part in this research, you will be agreeing to your information being seen by the study team only. Everybody who works with your information agrees to hold it in confidence.

Confidentiality may be broken if it is felt that there might be an issue regarding risk to yourself and/ or others. In this situation, the identity of the participant would be shared with the chief investigator who would decide on the relevant course of action in line with University of Sussex policies and procedures.

What will happen to the results of the research study?
When we have collected all the results for this study we will analyse them and then publish and present the results. Direct quotes may be used but these will not have your name or any other identifiable information attached. You will not be identified in any publication or presentation.

What are the possible benefits of taking part?
You will get a £20 gift card as a token of appreciation to thank you for completing the interview. If the interview is face-to-face, you will be offered refreshments. Also, providing feedback on a novel service allows healthcare professionals to shape and design services appropriately, to the benefit of future patients. Similarly, some people enjoy taking part in these types of studies where you can share your views and experiences. The findings will add to our knowledge about how to improve HIV and STI testing.

What are the potential disadvantages and risks of taking part in this study?
There are no risks or health implications to you by taking part in this study. It is possible that talking about this might make you feel upset. You can stop at any time without having to give a reason. You can also talk to us, someone else who you trust like friends or family, or your GP, if you have any concerns.

What are your choices about how your information is used?
You can stop being part of the study at any time, without giving a reason, and we will be able to delete your interview recording and data. However we will be unable to remove your questionnaire answers from the study as they include no identifiable information so the research team are unable to delete specific records. We need to manage your records in specific ways for the research to be reliable. This means that we won't be able to let you see or change the data we hold about you. If you would no longer like to take part in the research then please contact a member of the research team.

What if I would like to find out the results of the study?
If you would like to learn more about the findings of the study, please tick the appropriate box at the end of the questionnaire and enter a contact email. If you select this, at the end of the study we will email you a summary of the results. These will describe group findings and will not include any individual information.

For more information on how health researchers use information provided by patients please follow this link - https://www.sussex.ac.uk/library/researchdatamanagement/policies

Who is funding this research?

This research is sponsored by The University of Sussex and funded by the core department of Global Health at Brighton and Sussex Medical School. The Martin Fisher Foundation in collaboration with Brighton and Hove council has provided funding for the installation and maintenance of the five vending machines in Brighton and Hove. Unity Sexual Health Services, University Hospitals Bristol and Weston NHS Foundation Trust (UHBW) has provided funding for the installation and maintenance of the four vending machines in Bristol, North Somerset.

Where can you find out more about how your information is used?
If you have concerns about any aspects of the study, you should contact the research team using the contact information below. They will do their best to answer your questions. The contact details of the Data Protection Officer at the University of Sussex, and their data privacy policy, can be found https://www.sussex.ac.uk/about/website/privacy-and-cookies/privacy. The Universities of Sussex and Brighton have insurance for any liabilities resulting from this research. You can find out more about how we use your information at:

- www.hra.nhs.uk/information-about-patients/
- Our leaflet available at www.hra.nhs.uk/patientdataandresearch
- By asking one of the research team

- Dr Syra Dhillon syra.dhillon@nhs.net
  - Dr Jaime Vera J.Vera@bsms.ac.uk
  - Dr Jo Kesten jo.kesten@bristol.ac.uk
- By ringing us on 01273 523087
- University of Sussex Research Governance Officer at researchsponsorship@sussex.ac.uk

Thank you for taking the time to read this information sheet.

3. Interview CF

Patient Consent Form - Interview

Acceptability and feasibility of electronic vending machines for delivering HIV self-testing and STI self-sampling kits: a mixed methods study

Chief Investigator: Dr Jaime Vera
Version 5 16/05/22

	Please initial the box
I consent to being interviewed by the researcher.	
I confirm that I have read and understood the interview participant information sheet dated 16/05/22. I understand the principles, procedures and possible risks involved.	
I have had the chance to read the information and ask questions about the study, and am satisfied with the answers I have been given.	
I understand that taking part involves me taking part in an interview that will be audio recorded.	
I understand that any information I provide is confidential and that no information that I disclose will lead to the identification of any individual in the reports of the project, either by the researcher or by any other party.	
I consent to the processing of my personal information and data for the purposes of this research study. I understand that such information will be treated as confidential and handled in accordance with data protection legislation.	
I consent to the use of anonymised quotes in publications from the research.	
I understand that my participation in this study is voluntary and that I am free to withdraw at any time without being penalised or disadvantaged nor do I have to give a reason for doing so. The information I have already given will still be used in an anonymous form.	
I consent to my data being deposited in the UK Data Archive for reuse in future research and analysis. I understand that it will be fully anonymised before deposit.	
I wish to receive an update about the findings of this study and will provide my email address below.	
I understand that in exceptional circumstances, where a disclosure regarding your safety or that of others, the	

researcher will be legally required to pass this information onto an appropriate individual or agency.

I agree to take part in this research study.

If you would like an update on the findings of the study please provide a valid email address here;______________________.

Name of Participant Date Signature

Name of Researcher Date Signature

When completed: 1 copy for the participant; 1 copy for the researcher site file; *1 copy (original) to be kept in medical notes (delete if not applicable)*

4. Interview topic guide participant

Interview Topic Guide
Version 4 16/05/22

Acceptability and feasibility of electronic vending machines for delivering HIV self-testing and STI self-sampling kits: a mixed methods study

Gain verbal consent to start recording. Researchers should introduce themselves to the participant, briefly describe the study/interview timing and then allow sufficient time for reading the participant information sheet. After addressing any questions or concerns, written and verbal consent should be obtained.

1. Can you tell me about your previous experiences taking HIV tests?
    a. Have you ever had a test in a SH clinic/ with THT volunteers / self-sampling/ self-testing? How did you find the process? If you had never tested previously – have you ever thought about it? What made you decide not to? What would prompt you to test? What type of test would you choose?
2. What do you think about self-testing for HIV in general?
    a. What might be the benefits? The disadvantages? Is there anything that would worry you? In what circumstances do you think self-testing is most useful or most likely? Compared to self-sampling?
3. Can you tell me about your previous experiences taking STI tests?
    a. Have you ever had a test in a SH clinic/ at another health service / self-sampling? How did you find the process? Why did you choose those methods? if you have never tested – have you ever thought about it? What made you decide not to? What would prompt you to test? What type of test would you choose?
4. What do you think about self-sampling for STI in general?
    a. What might be the benefits? The disadvantages? Is there anything that would worry you? In what circumstances do you think self-sampling is most useful or most likely?
5. How do you feel about having a vending machine in a publicly available area?
    a. Which location did you use the vending machine?
    b. Why might it be a good idea? Why might it be a bad idea? Are there other places that would benefit from a similar vending machine or be more appropriate for it?
6. How do you feel about the machine dispensing both HIV and STI tests?
    a. Frequency of testing? What would you do if you developed symptoms?
7. Please tell me about further thoughts on your experience with the vending machine.
    a. Positive/negative? What was your motivation for using it? Would you use it again? Is there anything that would prompt you to use it in future?
8. Is there anything you would recommend to enable or encourage other people to use the vending machine and self-tests? Or a better alternative?
    a. Did you see any promotional material for it? Where might you expect to see this advertised?

Thank the participant and address any questions or concerns.

5. Interview topic guide stakeholder

Interview Topic Guide - stakeholders
Version 1 16/05/22

Acceptability and feasibility of electronic vending machines for delivering HIV self-testing and STI self-sampling kits: a mixed methods study

Gain verbal consent to start recording. Researchers should introduce themselves to the participant, briefly describe the study/interview timing and then allow sufficient time for reading the participant information sheet. After addressing any questions or concerns, written and verbal consent should be obtained.

1. Please describe venue
    a. Average customer demographic
    b. Your role within the institution
    c. How long has machine been here
2. Can you tell me about your experience of the vending machine at this site?
    a. Prompts:
    b. How have you been involved with the machine/ have you helped customers with the vending machine
    c. What is your perspective on having the machine in the xx
    d. How has having the machine at the xx impacted on your wok and the work of the xx generally?
    e. How have you seen other staff and/or customers relate to the machine?
    f. Have you seen any pros or cons to having the vending machine at the xx?
    g. Does it bring any advantages or disadvantages to the xx and/or to the customers and staff at the xx?
3. How do you think the vending machine is working – have you had any problems with it?
    a. Have customers needed help with it?
    b. Has it worked efficiently?
    c. Did anyone mention any problems with it?
    d. Who has had to resolve any problems with the machine?
    e. Have customers raised any problems with the machine and/or the self-tests to the staff?
    f. What do you think could be done to make the vending machine service more efficient or effective?
4. Can you suggest any other places that a vending machine could be sited?
    a. Follow-up: What do you think would be the pros and cons of different locations? What are the advantages and limitations of the vending machine in the xx?
5. Have you used the vending machine…?
    a. If Yes – how did you find the experience? What was your motivation for using it? Would you use it again?
    b. If No – what are your reasons for not using it? Is there anything that would prompt you to use it in future
6. What do you think about self-testing for HIV in general?
    a. What might be the benefits? The disadvantages? Is there anything that would worry you? In what circumstances do you think self-testing is most useful or most likely?
    b. As a venue, how do you feel about people doing tests here and getting a positive?
7. What do you think about self-testing for STIs in general?
    a. What might be the benefits? The disadvantages? Is there anything that would worry you? In what circumstances do you think self-testing is most useful or most likely?

Thank the participant and address any questions or concerns.
Stop recording

6. Coding tree

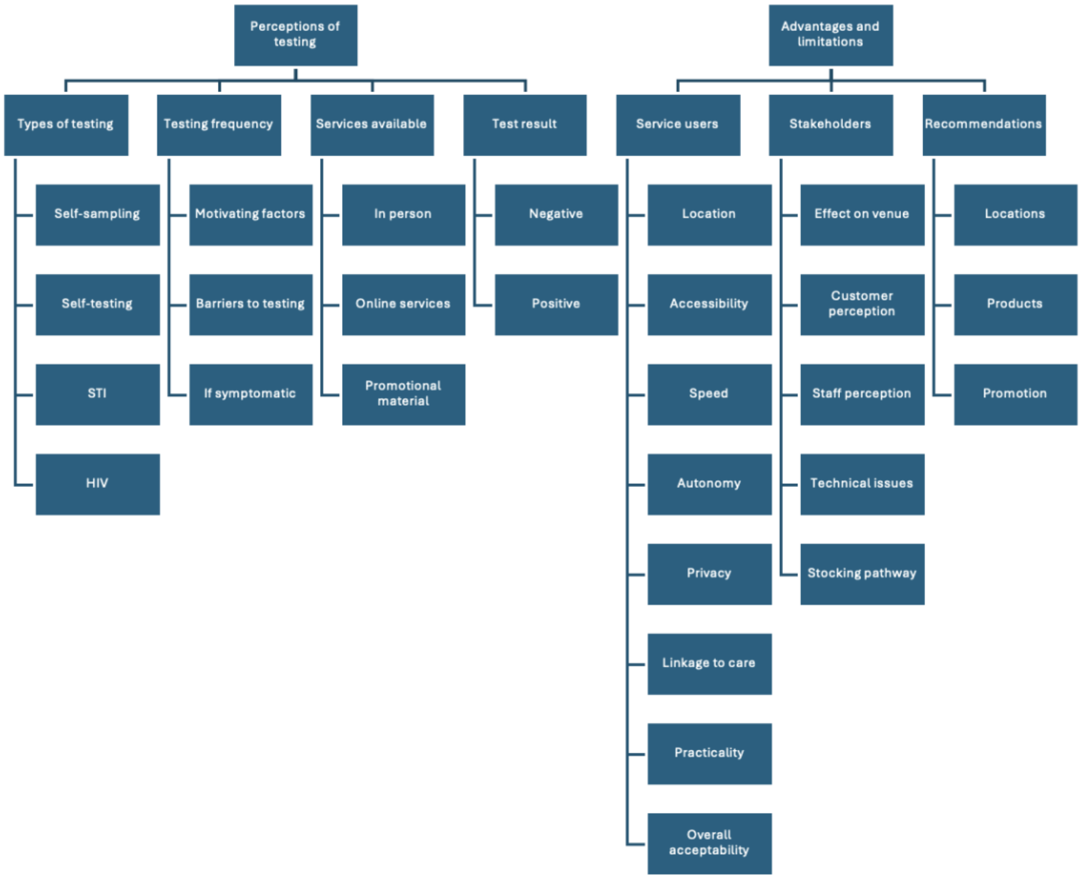

7. COREQ checklist

COREQ (COnsolidated criteria for REporting Qualitative research) Checklist

A checklist of items that should be included in reports of qualitative research. You must report the page number in your manuscript where you consider each of the items listed in this checklist. If you have not included this information, either revise your manuscript accordingly before submitting or note N/A.

Topic	Item No.	Guide Questions/Description	Reported on Page No.
**Domain 1: Research team and reflexivity**			
Personal characteristics			
Interviewer/facilitator	1	Which author/s conducted the interview or focus group?	6
Credentials	2	What were the researcher's credentials? E.g. PhD, MD	1
Occupation	3	What was their occupation at the time of the study?	6
Gender	4	Was the researcher male or female?	6
Experience and training	5	What experience or training did the researcher have?	6
Relationship with participants			
Relationship established	6	Was a relationship established prior to study commencement?	6
Participant knowledge of the interviewer	7	What did the participants know about the researcher? e.g. personal goals, reasons for doing the research	6
Interviewer characteristics	8	What characteristics were reported about the inter viewer/facilitator? e.g. Bias, assumptions, reasons and interests in the research topic	6
**Domain 2: Study design**			
Theoretical framework			
Methodological orientation and Theory	9	What methodological orientation was stated to underpin the study? e.g. grounded theory, discourse analysis, ethnography, phenomenology, content analysis	6
Participant selection			
Sampling	10	How were participants selected? e.g. purposive, convenience, consecutive, snowball	5
Method of approach	11	How were participants approached? e.g. face-to-face, telephone, mail, email	5
Sample size	12	How many participants were in the study?	7
Non-participation	13	How many people refused to participate or dropped out? Reasons?	7
Setting			
Setting of data collection	14	Where was the data collected? e.g. home, clinic, workplace	5
Presence of non-participants	15	Was anyone else present besides the participants and researchers?	6
Description of sample	16	What are the important characteristics of the sample? e.g. demographic data, date	7
Data collection			
Interview guide	17	Were questions, prompts, guides provided by the authors? Was it pilot tested?	5
Repeat interviews	18	Were repeat inter views carried out? If yes, how many?	6
Audio/visual recording	19	Did the research use audio or visual recording to collect the data?	6
Field notes	20	Were field notes made during and/or after the inter view or focus group?	6
Duration	21	What was the duration of the inter views or focus group?	5
Data saturation	22	Was data saturation discussed?	N/A
Transcripts returned	23	Were transcripts returned to participants for comment and/or	6

Topic	Item No.	Guide Questions/Description	Reported on Page No.
		correction?	
**Domain 3: analysis and findings**			
Data analysis			
Number of data coders	24	How many data coders coded the data?	6
Description of the coding tree	25	Did authors provide a description of the coding tree?	6
Derivation of themes	26	Were themes identified in advance or derived from the data?	6
Software	27	What software, if applicable, was used to manage the data?	6
Participant checking	28	Did participants provide feedback on the findings?	6
Reporting			
Quotations presented	29	Were participant quotations presented to illustrate the themes/findings? Was each quotation identified? e.g. participant number	8
Data and findings consistent	30	Was there consistency between the data presented and the findings?	10
Clarity of major themes	31	Were major themes clearly presented in the findings?	10
Clarity of minor themes	32	Is there a description of diverse cases or discussion of minor themes?	12

Developed from: Tong A, Sainsbury P, Craig J. Consolidated criteria for reporting qualitative research (COREQ): a 32-item checklist for interviews and focus groups. *International Journal for Quality in Health Care*. 2007. Volume 19, Number 6: pp. 349 – 357

Once you have completed this checklist, please save a copy and upload it as part of your submission. DO NOT include this checklist as part of the main manuscript document. It must be uploaded as a separate file.
